# Transylvania Medical Journal

**Published:** 1850-01

**Authors:** 


*Transylvania Medical Journal.*—A new periodical, with the above title, issued bi-monthly, has lately been commenced at Lexington, Ky., under the editorial management of Prof. Ethelbert L. Dudley, assisted by the medical Faculty of the Transylvania University. We have not, as yet, had the pleasure of receiving a copy.

By the way, having occasion to announce a new medical Journal, the inquiry is suggested, what has become of the Cleveland Medical Journal, advertised to appear many months ago?

